# Book Review: The Undoing Project: A Friendship that Changed Our Minds

**DOI:** 10.3389/fpsyg.2017.02211

**Published:** 2017-12-13

**Authors:** Gregory Bonn

**Affiliations:** Psychology, General Studies, King Fahd University of Petroleum and Minerals, Dhahran, Saudi Arabia

**Keywords:** decision making, collaborations, history of psychology, behavioral economics, cognitive biases

*The Undoing Project: A Friendship That Changed Our Minds* is best-selling author Lewis' (2017) account of the lives and deep collaboration between Daniel Kahneman and Amos Tversky. It tells the story of two brilliant psychologists, and distinctive personalities, who, through their research on cognitive biases, upended traditional notions of rationality in human thought that had previously served as the foundation for much economic and decision making theory. The importance of their research for fields as diverse as medicine and public health (e.g., Redelmeier and Tversky, 1990); decision making and economic policy (e.g., Thaler and Sunstein, 2008); as well as management and investment strategy earned Kahneman the Nobel Prize in Economic Sciences in 2002 (Tversky passed away in 1996).

Lewis (Moneyball, The Big Short, The Blind Side) is a story-teller with a unique ability to make complex ideas accessible and entertaining, and the extensive research he has done in preparing this book shows throughout. A years-long personal friendship with Dr. Kahneman, considerable cooperation from Tversky's remaining family members, and extensive interactions with the pair's many collaborators and colleagues allow him to provide color and insights into their personalities, personal histories, and relationships that no one else could. The reader, thus, is able to learn about Kahneman and Tversky's research and its many real-world implications through numerous illuminating and often surprising anecdotes. Lewis begins his chronicle, for example, with 30 pages examining how professional basketball teams evaluate prospective players, and the many ways in which this process is prone to error. We are not actually introduced to Kahneman until around page 50 of the text, where we get to know him through a recollection of his childhood experiences as a Jew in Nazi-occupied France, and his early forays into psychology as an officer in the Israeli army. Similarly, Tversky is introduced, some 40 pages later, as he is standing in line to apply to Hebrew University's newly formed psychology department; looking smart in his paratrooper's uniform and astounding compatriots with his wit. Using this kind of story-driven approach, Lewis provides a consistently compelling portrait of these two unique personalities and their formative influences, while chronicling their discovery of many, now well-known, biases in human reasoning. Often difficult to grasp concepts such as the availability heuristic, anchoring and adjustment, the base-rate fallacy, prospect theory, and loss aversion (e.g., Kahneman and Tversky, 1979; Kahneman et al., 1982; Kahnemann, 2011) are all set forth in narratives that even the most non-technical readers should find easy to digest.

True to Lewis' relaxed narrative approach, his protagonists don't actually meet until some 140 pages deep into the manuscript. There we witness the cocky mathematical genius Tversky being challenged by Kahneman over his presentation of a simple theory related to Bayesian decision making. Up to that point Tversky had more or less taken for granted, like most decision theorists of the era, that human thinking adhered to mathematical principles in some form. Kahneman; whom Lewis describes as a perpetual doubter, forever questioning everything, instinctively pounced on Tversky's overly theoretical description of human thought. From Kahneman's perspective, humans make decisions based on impressions; math need not be involved. Rather than starting from real behavior, decision theorists were, in Kahneman's view, simply creating mathematical models and attempting to interpret reality through those models. Apparently, for Tversky this was something of a revelation. From that time on, the two began to spend more and more time together, bouncing ideas around and gradually developing what would become a decades-long friendship and collaboration.

Lewis' work is, at heart, the story of Kahneman and Tversky's relationship. For many years the two were practically inseparable, spending hours together most days, simply exploring all manner of ideas, and laughing. The two complemented each other in important ways: Tversky freed the naturally insecure Kahneman to experience a level of confidence and joy; while Kahneman pushed the hyper-confident Tversky to question ideas and see different perspectives in a manner that others could not. As described by Lewis, they worked so closely together that neither had any real conception of where their ideas came from. In this sense, theirs was a true collaboration; their work together became much more than either could lay claim to individually. Their story takes its tragic turns however: The two take up positions at different universities and, although they continue to collaborate, over time, jealousy and resentment creep into their exchanges. By the time of Tversky's early death from cancer, their friendship was just a faint reflection of its previous form.

In sum, aside from being just generally entertaining, *The Undoing Project* will be a worthwhile and enjoyable read for anyone with an interest in decision making, the research process and the nature of collaboration. The interested reader is rewarded on many levels by Lewis' work: It has engaging portrayals of history, war, and survival. It tells the story of a unique friendship between two exceptional individuals. And, it details important psychological findings as well as their broader implications in lively anecdotal form. Prospective graduate students and others who are considering a career in academia can learn much about university life and the nature of collaboration from this work. Others, with even a passing interest in psychology and human nature, should find the many stories and insights presented here to be compelling reading as well.

## Author contributions

The author confirms being the sole contributor of this work and approved it for publication.

### Conflict of interest statement

The author declares that the research was conducted in the absence of any commercial or financial relationships that could be construed as a potential conflict of interest.
